# FAT3 Mutation Is Associated With Tumor Mutation Burden and Poor Prognosis in Esophageal Cancer

**DOI:** 10.3389/fonc.2021.603660

**Published:** 2021-03-19

**Authors:** Zixin Guo, Xin Yan, Congkuan Song, Qingwen Wang, Yujin Wang, Xiao-Ping Liu, Jingyu Huang, Sheng Li, Weidong Hu

**Affiliations:** ^1^ Department of Thoracic Surgery, Zhongnan Hospital of Wuhan University, Wuhan, China; ^2^ Department of Biological Repositories, Zhongnan Hospital of Wuhan University, Wuhan, China; ^3^ Department of Urology, Zhongnan Hospital of Wuhan University, Wuhan, China; ^4^ Hubei Key Laboratory of Tumor Biological Behaviors, Hubei Cancer Clinical Study Center, Wuhan, China; ^5^ Human Genetics Resource Preservation Center of Hubei Province, Wuhan, China

**Keywords:** FAT3, esophageal cancer, tumor mutation burden, prognostic marker, bioinformatics

## Abstract

**Objective:**

To explore the mutated genes in esophageal cancer (ESCA), and evaluate its relationship with tumor mutation burden (TMB) and prognosis of ESCA, and analyze the advantages of FAT3 as a potential prognostic marker in ESCA.

**Methods:**

The somatic mutation landscape was analyzed according to ESCA samples from the TCGA and ICGC database. The differences of TMB between mutant type and wild type of frequently mutated genes were compared by Mann-Whitney U test. The association of gene mutations with prognosis was analyzed by Kaplan-Meier method. The relative abundance of 22 tumor-infiltrating lymphocyte subsets in ESCA was calculated by CIBERSORT algorithm.

**Results:**

FAT3 was a high frequency mutation in both TCGA and ICGC samples from the somatic mutation landscape. Then, the mutation type of FAT3 had significantly higher TMB in patients with ESCA compared the wild type (*P*<0.05). Meanwhile, the prognosis of FAT3 mutation type was significantly worse in patients with ESCA(*P*<0.05), and the FAT3 mutation status might be an independent factor for prognosis of patients with ESCA (HR: 1.262–5.922, P=0.011). The GSEA analysis revealed the potential mechanism of FAT3 mutation on the occurrence and development of ESCA. Finally, naive B cells were significantly enriched in FAT3 mutation samples of the ESCA microenvironment (*P*<0.05).

**Conclusions:**

FAT3 mutation is related to TMB and poor prognosis in ESCA. FAT3 mutation may be a prognostic marker of ESCA, and reveal the potential mechanism of FAT3 mutation on ESCA.

## Introduction

Esophageal cancer (ESCA) is a common malignant tumor of the digestive tract in the world. It has the characteristics of high malignancy and poor prognosis. The main pathological types of ESCA include esophageal squamous cell carcinoma (ESCC) which is more common in Asian countries, and esophageal adenocarcinoma (EADC) which is more common in Western countries (1–3). ESCA is mainly characterized by progressive dysphagia, and most patients diagnosed with esophageal cancer have entered the advanced stage. Despite some progress in ESCA surgical techniques, chemotherapy and radiotherapy protocols, and perioperative management, the 5-year survival rate of patients with ESCA is about 19%, which is a serious threat to human life and health (4, 5).

Tumor mutation burden (TMB) is the total number of somatic gene variants detected per million bases of genomic DNA, including base substitutions, insertions, or deletions. Tumor cells can produce many specific mutations at the genetic level, and every 150 non-synonymous mutations may produce one to two neoantigens, which can be recognized by the autoimmune system, thus activating T cells and causing immune response (6–8). The more non-synonymous mutations, the more neoantigens will be recognized by the autoimmune system, and the stronger the autoimmune effect can be caused. Therefore, the higher TMB, the easier it will benefit from immunosuppressive therapy (9, 10). Several clinical studies have demonstrated that TMB can predict the efficacy of immunotherapy for solid tumors, such as melanoma (11), bladder cancer (12), small cell lung cancer (13).

In this study, the gene mutations of ESCA were analyzed by using the single-nucleotide variants (SNV)data from The Cancer Genome Atlas (TCGA) and International Cancer Genome Consortium (ICGC), and then the common high-frequency mutation genes were screened out. Then, we explored the association of high-frequency mutant genes with TMB and overall survival, and conducted gene enrichment analysis on mutant genes closely related to prognosis. Finally, the association of key mutant genes with immune infiltration was explored. This study may identify a marker for ESCA, which had the potential as a target for immunotherapy.

## Materials and Methods

### Data Resources

The SNV data of ESCA was downloaded from the TCGA database(https://portal.gdc.cancer.gov/), containing 184 samples. The simple somatic mutation data of ESCA was downloaded from the ICGC database (https://dcc.icgc.org/releases/current/Projects/ESCA-CN), containing 298 samples from China. The clinical information data of ESCA was downloaded from the TCGA.

The clinical information data were collated to obtain 144 samples, including survival time, survival status, age, gender, TNM, and stage.

### Identification of Frequently Mutated Genes

MAF files were conducted by VarScan for somatic variants for ESCA samples from the TCGA database, and waterfall plots were used to visualize somatic variants for ESCA samples by maftools package (14). TSV files were annotated by hg19 reference genome and visualized using GenVisR package for somatic variants for ESCA samples from the ICGA database. Then, the top 30 high-frequency mutated genes were selected by ranking the frequency of gene mutations from high to low in the TCAG database and ICGC database. The common frequently mutated genes were intersected that were both of the top 30 high-frequency mutated genes in the TCAG database and ICGC database.

### Relationship Between Gene Mutation and TMB and Prognosis

The whole-exome sequencing (WES) data was used to calculate the TMB score in esophageal cancer from TCGA database. The total number of non-synonymous somatic variants was divided by exome size to calculate the TMB score from TCGA database (15). The differences of TMB between mutant type and wild type of frequently mutated genes were compared to explore the relationship between gene mutation and TMB. The association of gene mutations with survival prognosis was analyzed by Kaplan-Meier method. Univariate and multivariate Cox regression was analyzed to prove that FAT3 was an independent factor. The FAT3 mutation status and some clinical indicators such as age, gender, stage, TMB, and other genes mutation status were included in the analysis.

### Gene Set Enrichment Analysis

The transcriptome data from TCGA database was used in gene set enrichment analysis (GSEA). According to the FAT3 mutation status, the samples were divided into mutation and wild groups. The GSEA was conducted by GSEA software (version 4.0.3) from Broad Institute (16).The gene set “c2.all.v7.1.symols.gmt” in MsigDB database was used as reference gene set. Permutations for each analysis were set as 1000 times. Pathways with normal *P*-value <0.01 were considered significantly enriched.

### Association of Gene Mutations With Tumor-infiltrating Immune

The relative abundance of 22 tumor-infiltrating lymphocyte subsets in ESCA patients with different FAT3 mutation status were calculated using CIBERSORT algorithm (17). The relative abundance of tumor-infiltrating immune was calculated after correction of gene expression, and samples with *P*-value<0.05 were included in the study. The bar plot and violin plot were used to show the association of gene mutations with tumor-infiltrating immune.

### Statistical Analysis

The relationship between gene mutation and TMB were analyzed by using GraphPad Prism software (version 8.0). The relationship between gene mutation and TMB was analyzed by Mann-Whitney U test. Univariate and Multivariate Cox analysis were performed with IBM SPSS Statistics (version 20), and the method “forwaed:LR” was used in Multivariate Cox analysis. Other analyses, such as mutation information analysis, survival analysis, and tumor-infiltrating immune analysis, were performed using R software (version 3.6.1). The survival curves were drawn by Kaplan-Meier method, and Log-rank test was used to evaluate the survival analysis. *P*-values<0.05 were considered significant.

## Results

### Identification of Frequently Mutated Genes in ESCA

**Figure 1:**
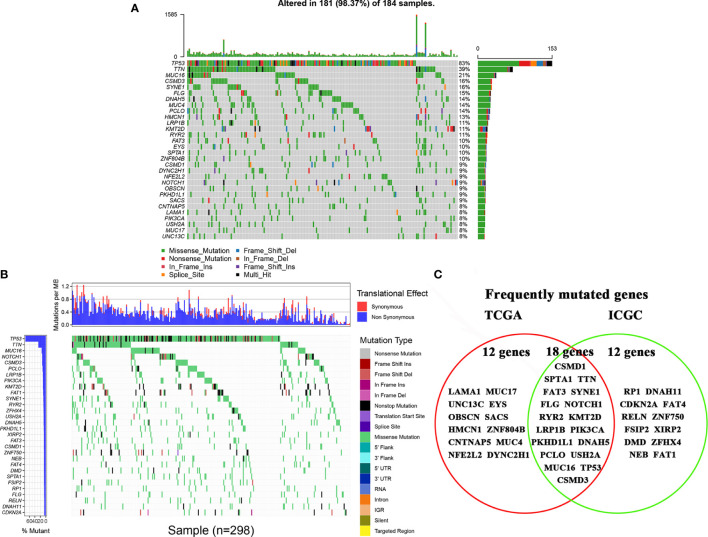
Landscapes of frequently mutated genes in esophageal cancer. **(A)** Oncoplot displaying the landscapes of frequently mutated genes in ESCA from the TCGA database. Genes are ordered according to their mutation frequency (left panel), and different mutation types were presented as indicated by the annotation bar (bottom). **(B)** Waterfall plot displaying the landscapes of frequently mutated genes in ESCA from the ICGC database. Genes are ordered according to their mutation frequency (left panel), and different mutation types were presented as indicated by the annotation bar (right panel). **(C)** Venn diagram displaying the common frequently mutated genes that were both of the top 30 high-frequency mutated genes in the TCAG database and ICGC database.

The top 30 high-frequency mutant genes from the TCGA database were showed in **Figure 1A**. TP53, TTN, MUC16, CSMD3, and SYNE1 were the five genes with the highest mutation frequency in the TCGA database. Meanwhile, the top 30 high-frequency mutant genes from the ICGC database were showed in **Figure 1B**. TP53, TTN, MUC16, NOTCH1, and CSMD3 were the five genes with the highest mutation frequency in the ICGC database. Finally, we obtained 18 frequently mutated genes in ESCA that were represented in both TCGA and ICGA database, containing CSMD1, SPTA1, TTN, FAT3, SYNE1, FLG, NOTCH1, RYR2, KMT2D, LRP1B, PIK3CA, PKHD1L1, DNAH5, PCLO, USH2A, MUC16, TP53, and CSMD3 (**Figure 1C**).

### Relationship Between Gene Mutation and TMB and Prognosis

**Figure 2:**
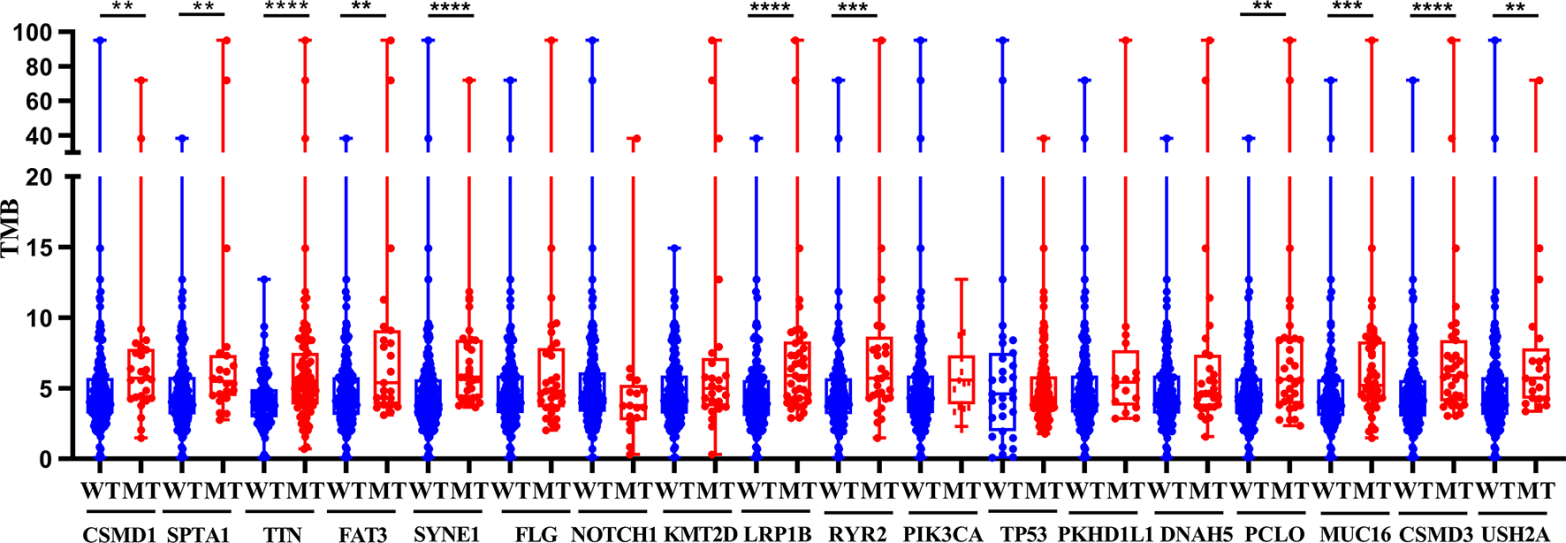
Gene mutations are associated with TMB. Compared with the wild type of genes, the mutation type of CSMD1, SPTA1, TTH, FAT3, SYNE1, LRP1B, RYR2, PCLO, MUC16, CSMD3, and USH2A had significantly higher TMB in patients with ESCA. **p < 0.01; ***p < 0.001; ****p<0.0001. WT, wild type; MT, mutant type.

**Figure 3:**
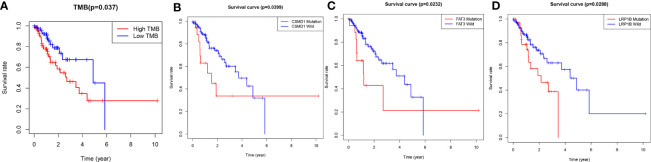
Gene mutations are associated with clinical prognosis. **(A)** The high-TMB group was related to a negative prognosis in ESCA. **(B–D)** The association of gene mutations with survival prognosis was analyzed by Kaplan-Meier method. A total of 144 samples containing complete clinical information were included. *P*-values<0.05 were considered significant. WT, wild type; MT, mutant type.

The TMB score was calculated based on SNV data of ESCA from the TCGA database. Compared with the wild type of genes, the mutation type of CSMD1, SPTA1, TTH, FAT3, SYNE1, LRP1B, RYR2, PCLO, MUC16, CSMD3, and USH2A had significantly higher TMB in patients with ESCA (*P*<0.05, **Figure 2**). Based on the median of TMB score, the samples were divided into high-TMB group and low-TMB group. Based on the Kaplan-Meier analysis, the high-TMB group was related to a negative prognosis in ESCA (*P*<0.05, **Figure 3A**). For mutant genes with significantly higher TMB, we conducted survival analysis and found that the prognosis of CSMD1, FAT3, and LRP1B mutation type were significantly worse in patients with ESCA (P<0.05, **Figures 3B–D**). The results of survival analysis by other gene mutation status were not statistically significant (P>0.05, **Supplementary Figures 1A–H**). The CSMD1, FAT3, and LRP1B mutation status, and some clinical indicators such as age, gender, stage, and TMB were included in the Cox regression analysis. The FAT3 mutation status was statistically significance in Cox regression analysis (**Table 1**), which might be an independent prognosis factor for patients with ESCA (HR: 1.262-5.922, P=0.011).

**Table 1:** Univariate and multivariate Cox analysis of esophageal cancer patients by the IBM SPSS Statistics (version 20).

Factors	Univariate		Multivariate	
	HR (95%CI)	P-value	HR (95%CI)	P-value
Age (year)(<60,≥60)	1.326(0.712–2.471)	0.374		
Gender (male, female)	0.831(0.325–2.125)	0.7		
Stage (stage I and II, stage III and IV)	3.743(1.929–7.261)	<0.001	3.992(2.043–7.800)	<0.001
TMB (low, high)	1.971(1.031–3.767)	0.04	–	0.257
LRP1B (wide, mutant)	2.073(1.063–4.044)	0.032	–	0.409
FAT3 (wide, mutant)	2.353(1.102–5.026)	0.027	2.734(1.262–5.922)	0.011
CSMD1 (wide, mutant)	2.169(1.019–4.614)	0.044	–	0.551

### GSEA of FAT3 Mutation

**Figure 4:**
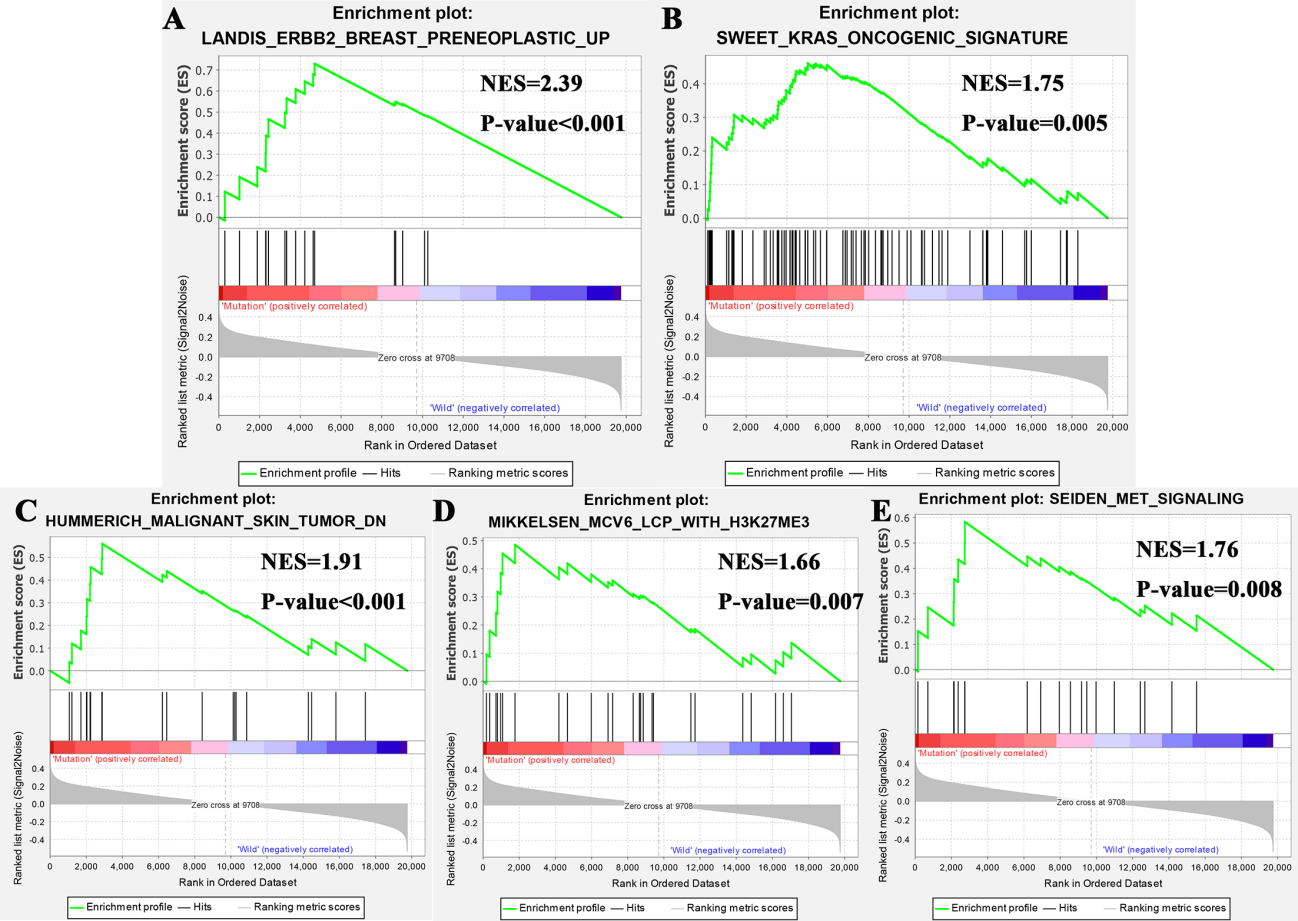
Significantly enriched pathways associated with FAT3 mutation. GSEA was performed with TCGA to explore the function role of FTA3 mutation. The GSEA analysis showed that samples with FAT3 mutation enriched in **(A)** ERBB2 Breast Preneoplastic UP, **(B)** Kras Oncogenic Signature, **(C)** “Malignant Skin Tumor DN, **(D)** MCV6 LCP With H3K27ME3 pathway, and **(E)** MET Signaling pathway. NES, normalized enrichment score.

Gene enrichment analysis was performed with TCGA to explore the function role of FTA3 mutation. The results of GSEA analysis showed that samples with FAT3 mutation enriched in “ERBB2 Breast Preneoplastic UP”, “Kras Oncogenic Signature”, “Malignant Skin Tumor DN”, “MCV6 LCP With H3K27ME3”, and “MET Signaling” (**Figure 4**). These pathways revealed the potential mechanism of FAT3 mutation on the occurrence and development of ESCA, and provided support for further exploring the role of FAT3 mutation in tumors.

### Association of FAT3 Mutation With Tumor-infiltrating Immune

**Figure 5:**
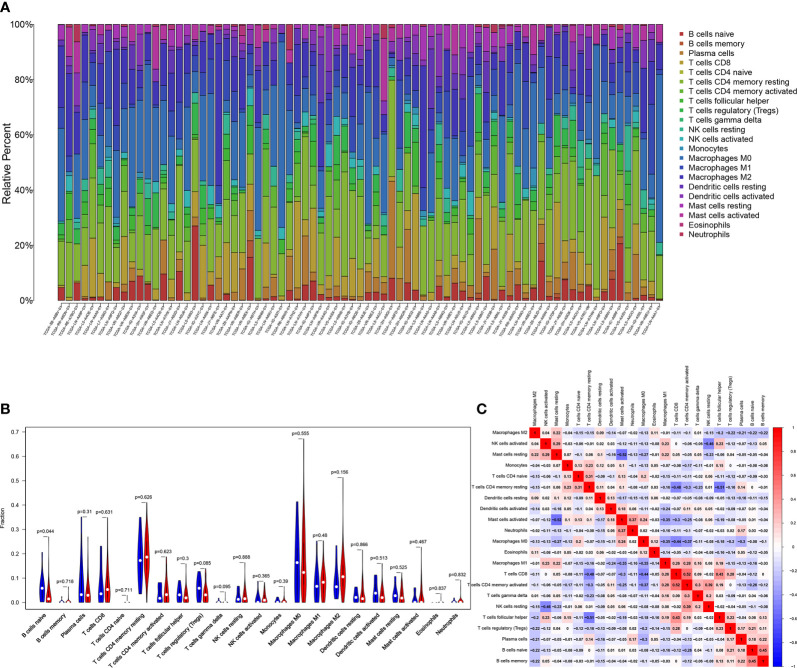
Association of FAT3 Mutation with Tumor-infiltrating Immune. **(A)** Stacked bar chart shows the proportion of 22 immune cells in each samples of ESCA. **(B)** Violin plot displays the differentially infiltrated immune cells between FAT3-mutant groups and FAT3-wild group. Blue color: FAT3-wild group; Red color: FAT3-mutant group. **(C)** Correlation matrix of immune cell proportions. Red color: positive correlation; Blue color: negative correlation.

Research has shown that TMB may be a potential marker for predicting the efficacy of immunotherapy (18), therefore, we explored the relationship between FAT3 mutation and tumor-infiltrating immune in ESCA. The immune infiltration of the ESCA microenvironment was calculated using CIBERSORT algorithm, and the Stacked bar showed the proportion of 22 immune cells in each samples of ESCA (**Figure 5A**). Compared with the FAT3 wild type, naive B cells were significantly enriched in FAT3 mutation type of the ESCA microenvironment (*P*<0.05, **Figure 5B**). Moreover, activated memory CD4^+^ T cells had the strongest positive correlation with CD8^+^ T cells, and activated Mast cells had the strongest negative correlation with resting Mast cells from the correlation matrix (**Figure 5C**). For naive B cells, the strongest positive correlation was the memory B cells and the strongest negative correlation was activated memory CD4^+^ T cells (**Figure 5C**).

## Discussion

In this study, the somatic mutation landscape was analyzed according to ESCA samples from the TCGA database containing 184 samples and the ICGC database containing Chinese samples. FAT3 was a high frequency mutation in both TCGA and ICGC samples from the somatic mutation landscape. Then, the mutation type of FAT3 had significantly higher TMB in patients with ESCA compared the wild type. Meanwhile, the prognosis of FAT3 mutation type was significantly worse in patients with ESCA, and the FAT3 mutation status was statistically significance in Cox regression analysis, which might be an independent prognosis factor for patients with ESCA. The GSEA analysis revealed the potential mechanism of FAT3 mutation on the occurrence and development of ESCA, and provided support for further exploring the role of FAT3 mutation in tumors. Finally, naive B cells were significantly enriched in FAT3 mutation samples of the ESCA microenvironment.

FAT3 was a cadherin gene located on chromosome 11q14.3-q21. Experiments on homologs of rodent FAT3 showed that its mRNA was highly expressed in embryonic rat brain, but its expression was relatively low in adult brain tissue (19). In mice, the expression of FAT3 was limited to the developing central nervous system, with the highest expression in olfactory bulb and retina (20). FAT3 might control the polarized development of tissues through cytoskeleton, thereby affecting the emergence of asymmetric cell morphology during retinal development (21). The point mutation of FAT3 can cause pancreatic tumor in human cancer (22). Studies have shown that FAT3 was a key mutation gene in multiple tumors, including ovarian cancer, breast cancer (23), lung adenocarcinoma (24), and pancreatic acinar cell carcinoma (25). Studies have reported that the whole genome expression of lung tumor cell populations of transgenic mice was analyzed by laser capture microdissection, and it was found that FAT3 mRNA was significantly down-regulated in lung adenocarcinoma. It was speculated that such molecular switches may promote the transformation of epithelial dysplasia into lung glands cancer (26). FAT3 was relatively less studied and was thought to participate in the development of human cancer through a pathway similar to that of the Ena/VASP proteins (22). These studies might indicate that FAT3 mutation played an important role in the occurrence and development of tumors.

ESMO collected the TMB levels of 104,814 patients with 30 kinds of solid tumors, and identified the highest level of TMB, namely cutaneous malignant melanoma, followed by non-small cell lung cancer and other squamous cell carcinoma (27). The higher the level of TMB, the better the effect of immunotherapy (10). And this was confirmed in patients with high levels of TMB in melanoma and lung squamous carcinoma (11). The mutation type of FAT3 had significantly higher TMB in patients with ESCA compared the wild type in this study. The high-TMB group was related to a negative prognosis in ESCA from the Kaplan-Meier analysis. These results may suggest that immunotherapy for ESCA patients with high TMB will obtain higher benefits compared with patients with low TMB.

GSEA analysis showed that samples with FAT3 mutation enriched in “ERBB2 Breast Preneoplastic UP”, “Kras Oncogenic Signature”, “Malignant Skin Tumor DN”, “MCV6 LCP With H3K27ME3”, and “MET Signaling”. The “ERBB2 Breast Preneoplastic UP” showed that the TGF-beta pathway was intrinsically suppressed in ErbB2/Neu tumors *via* a mechanism involving loss of TGF-beta-Receptor-I/ALK5 (28). “Kras Oncogenic Signature” revealed that FAT3 mutation may affect ESCA through KRAS pathway (29). “Malignant Skin Tumor DN” showed that the mechanism of FAT3 mutation in the occurrence and development of ESCA was related to that of malignant skin (30). “MCV6 LCP With H3K27ME3” was related to cell differentiation (31). MET signaling pathway played an important role in cell migration, apoptosis, proliferation, and differentiation. It can promote tumor cells to form a more aggressive cell phenotype to avoid immunity, and enhance the survival, and invasion ability of tumor cells (32, 33).

At present, several studies have confirmed that there are differences in the expression of immune cells in tumor tissues. The proportion of M1 macrophages was higher in nasopharyngeal carcinoma, while memory B cells and resting memory CD4^+^ T cells were relatively lower (34). The proportion of M0 macrophages and activated memory CD4^+^ T cells was higher, while neutrophils and monocytes were lower in prostate cancer tissues (35). The expression of activated memory CD4^+^ T cells, M0 macrophages, and M1 macrophages in colorectal cancer was higher than that in normal tissues (36). In this study, naive B cells were significantly enriched in FAT3 mutation type of the ESCA microenvironment compared with the FAT3 wild type. For naive B cells, the strongest positive correlation was the memory B cells and the strongest negative correlation was activated memory CD4 T cells. B cells may play an important role in tumor immunity of ESCA. More than 90% of gastroesophageal adenocarcinoma tumor tissues had more tumor infiltrating B cells (37). Studies had found that there were a variety of memory B cells in pancreatic cancer tissues, such as IgG ^+^, IgG2a/b ^+^, IgA ^+^ memory B cells, and B cells were not conducive to tumor progression (38). Shi et al. (39) found that margin-infiltrating B lymphocytes at the edge of cancer presented atypical memory phenotype (IgD^−^IgG^+^CD27^−^CD38^−^). Margin-infiltrating B lymphocytes could secrete IFN-γ and IL-12p40, promote Th1 immune response and exert anti-tumor activity. Nielsen et al. (40) showed that CD20^+^ tumor-infiltrating lymphocytes similarly had an atypical memory phenotype (CD27^−^) in ovarian cancer and that the cells highly expressed antigen-presenting molecules (MHC-I, MHC-II, CD40, CD80, CD86) and co localized with CD8^+^ T cells, suggesting that it presented tumor antigens and activated tumor killer T cells, resulting in tumor suppression. CD4^+^ T lymphocytes can not only help CD8^+^ cytotoxic T lymphocytes, but also help NK cells to kill tumors. For example, it had been found that CD4^+^ T lymphocytes were better than CD8^+^ T lymphocytes in rejecting solid tumors in *in vitro* experiments (41, 42). CD4^+^ T lymphocytes can both inhibit tumor growth and promote tumor growth (43, 44). The above research results indicated that CD4^+^ T lymphocytes were complex and changeable in the anti-tumor cell immunity. These results may indicate the potential mechanism of immune microenvironment affecting ESCA.

This study also has some limitations. Firstly, there are few FAT3 mutation-type samples compared with wild-type samples. secondly, due to technical limitations, no clinical samples were collected to verify the results.

## Conclusions

In conclusion, through the analysis of somatic mutation data in the TCGA and ICGC databases, FAT3 mutation was a high frequency mutation gene in ESCA. Then, the association of FAT3 mutation with TMB and prognosis was obtained. FAT3 mutation was an independent risk factor in ESCA. Furthermore, the GSEA of FAT3 mutation and the relationship between FAT3 mutation and tumor-infiltrating immune were explored. These results indicated that FAT3 mutation was a prognostic marker of ESCA, and revealed the potential mechanism of FAT3 mutation on ESCA.

## Data Availability Statement

The original contributions presented in the study are included in the article/**Supplementary Material**. Further inquiries can be directed to the corresponding authors.

## Author Contributions

WH and SL conceived and designed the study. ZG, XY, and CS performed the analysis procedures. XY, ZG, QW, and YW analyzed the results. JH, X-PL, and SL contributed analysis tools. ZG, and XY contributed to the writing of the manuscript. All authors have reviewed the manuscript. All authors contributed to the article and approved the submitted version.

## Funding

The present study was supported by Zhongnan Hospital of Wuhan University Science, Technology and Innovation Seed Fund, Project znpy2019088.

## Conflict of Interest

The authors declare that the research was conducted in the absence of any commercial or financial relationships that could be construed as a potential conflict of interest.

## Supplementary Material

Supplementary Figure 1 Gene mutations aren’t associated with clinical prognosis. **(A–H)** The association of gene mutations with survival prognosis was analyzed by Kaplan-Meier method. A total of 144 samples containing complete clinical information were included. *P*-values<0.05 were considered significant. WT, wild type; MT, mutant type.

## References

[1] Pickens A Orringer MB. Geographical distribution and racial disparity in esophageal cancer. Ann Thorac Surg (2003) 76(4):S1367–9. 10.1016/s0003-4975(03)01202-5 14530066

[2] Ferlay J Soerjomataram I Dikshit R Eser S Mathers C Rebelo M. Cancer incidence and mortality worldwide: sources, methods and major patterns in GLOBOCAN 2012. Int J Cancer (2015) 136(5):E359–86. 10.1002/ijc.29210 25220842

[3] Macedo-Silva C Miranda-Gonçalves V Henrique R Jerónimo C Bravo I. The Critical Role of Hypoxic Microenvironment and Epigenetic Deregulation in Esophageal Cancer Radioresistance. Genes (Basel) (2019) 10(11):927. 10.3390/genes10110927 PMC6896142 31739546

[4] Domper AM Ferrández AÁ Lanas AÁ. Esophageal cancer: Risk factors, screening and endoscopic treatment in Western and Eastern countries. World J Gastroenterol (2015) 21(26):7933–43. 10.3748/wjg.v21.i26.7933 PMC4499337 26185366

[5] Siegel RL Miller KD Jemal A. Cancer statistics, 2019. CA Cancer J Clin (2019) 69(1):7–34. 10.3322/caac.21551 30620402

[6] Yarchoan M Hopkins A Jaffee EM. Tumor Mutational Burden and Response Rate to PD-1 Inhibition. N Engl J Med (2017) 377(25):2500–1. 10.1056/NEJMc1713444 PMC6549688 29262275

[7] Mansfield AS Ren H Sutor S Sarangi V Nair A Davila J. Contraction of T cell richness in lung cancer brain metastases. Sci Rep (2018) 8(1):2171. 10.1038/s41598-018-20622-8 29391594 PMC5794798

[8] Gubin MM Artyomov MN Mardis ER Schreiber RD. Tumor neoantigens: building a framework for personalized cancer immunotherapy. J Clin Invest (2015) 125(9):3413–21. 10.1172/JCI80008 PMC4588307 26258412

[9] Matsushita H Vesely MD Koboldt DC Rickert CG Uppaluri R Magrini VJ. Cancer exome analysis reveals a T-cell-dependent mechanism of cancer immunoediting. Nature (2012) 482(7385):400–4. 10.1038/nature10755 PMC3874809 22318521

[10] Rooney MS Shukla SA Wu CJ Getz G Hacohen N. Molecular and genetic properties of tumors associated with local immune cytolytic activity. Cell (2015) 160(1-2):48–61. 10.1016/j.cell.2014.12.033 25594174 PMC4856474

[11] Snyder A Makarov V Merghoub T Yuan J Zaretsky JM Desrichard A. Genetic basis for clinical response to CTLA-4 blockade in melanoma. N Engl J Med (2014) 371(23):2189–99. 10.1056/NEJMoa1406498 PMC4315319 25409260

[12] Rosenberg JE Hoffman-Censits J Powles T van der Heijden MS Balar AV Necchi A. Atezolizumab in patients with locally advanced and metastatic urothelial carcinoma who have progressed following treatment with platinum-based chemotherapy: a single-arm, multicentre, phase 2 trial. Lancet (2016) 387(10031):1909–20. 10.1016/S0140-6736(16)00561-4 PMC5480242 26952546

[13] Hellmann MD Callahan MK Awad MM Calvo E Ascierto PA Atmaca A. Tumor Mutational Burden and Efficacy of Nivolumab Monotherapy and in Combination with Ipilimumab in Small-Cell Lung Cancer. Cancer Cell (2018) 33(5):853–61.e4. 10.1016/j.ccell.2018.04.001 29731394 PMC6750707

[14] Mayakonda A Lin DC Assenov Y Plass C Koeffler HP. Maftools: efficient and comprehensive analysis of somatic variants in cancer. Genome Res (2018) 28(11):1747–56. 10.1101/gr.239244.118 PMC6211645 30341162

[15] Chalmers ZR Connelly CF Fabrizio D Gay L Ali SM Ennis R. Analysis of 100,000 human cancer genomes reveals the landscape of tumor mutational burden. Genome Med (2017) 9(1):34. 10.1186/s13073-017-0424-2 28420421 PMC5395719

[16] Subramanian A Tamayo P Mootha VK Mukherjee S Ebert BL Gillette MA. Gene set enrichment analysis: a knowledge-based approach for interpreting genome-wide expression profiles. Proc Natl Acad Sci U S A (2005) 102(43):15545–50. 10.1073/pnas.0506580102 PMC1239896 16199517

[17] Newman AM Liu CL Green MR Gentles AJ Feng W Xu Y. Robust enumeration of cell subsets from tissue expression profiles. Nat Methods (2015) 12(5):453–7. 10.1038/nmeth.3337 PMC4739640 25822800

[18] Le DT Uram JN Wang H Bartlett BR Kemberling H Eyring AD. PD-1 Blockade in Tumors with Mismatch-Repair Deficiency. N Engl J Med (2015) 372(26):2509–20. 10.1056/NEJMoa1500596 PMC4481136 26028255

[19] Mitsui K Nakajima D Ohara O Nakayama M. Mammalian fat3: a large protein that contains multiple cadherin and EGF-like motifs. Biochem Biophys Res Commun (2002) 290(4):1260–6. 10.1006/bbrc.2002.6338 11811999

[20] Nagae S Tanoue T Takeichi M. Temporal and spatial expression profiles of the Fat3 protein, a giant cadherin molecule, during mouse development. Dev Dyn (2007) 236(2):534–43. 10.1002/dvdy.21030 17131403

[21] Krol A Henle SJ Goodrich LV. Fat3 and Ena/VASP proteins influence the emergence of asymmetric cell morphology in the developing retina. Development (2016) 143(12):2172–82. 10.1242/dev.133678 PMC4920175 27122175

[22] Zhang X Liu J Liang X Chen J Hong J Li L. History and progression of Fat cadherins in health and disease. Onco Targets Ther (2016) 9:7337–43. 10.2147/OTT.S111176 PMC5138043 27942226

[23] Longacre M Snyder NA Housman G Leary M Lapinska K Heerboth S. A Comparative Analysis of Genetic and Epigenetic Events of Breast and Ovarian Cancer Related to Tumorigenesis. Int J Mol Sci (2016) 17(5):759. 10.3390/ijms17050759 PMC4881580 27213343

[24] Cai W Zhou D Wu W Tan WL Wang J Zhou C. MHC class II restricted neoantigen peptides predicted by clonal mutation analysis in lung adenocarcinoma patients: implications on prognostic immunological biomarker and vaccine design. BMC Genomics (2018) 19(1):582. 10.1186/s12864-018-4958-5 30075702 PMC6090856

[25] Furukawa T Sakamoto H Takeuchi S Ameri M Kuboki Y Yamamoto T. Whole exome sequencing reveals recurrent mutations in BRCA2 and FAT genes in acinar cell carcinomas of the pancreas. Sci Rep (2015) 5:8829. 10.1038/srep08829 25743105 PMC4351513

[26] Rohrbeck A Borlak J. Cancer genomics identifies regulatory gene networks associated with the transition from dysplasia to advanced lung adenocarcinomas induced by c-Raf-1. PLoS One (2009) 4(10):e7315. 10.1371/journal.pone.0007315 19812696 PMC2754338

[27] Chan TA Yarchoan M Jaffee E Swanton C Quezada SA Stenzinger A. Development of tumor mutation burden as an immunotherapy biomarker: utility for the oncology clinic. Ann Oncol (2019) 30(1):44–56. 10.1093/annonc/mdy495 30395155 PMC6336005

[28] Landis MD Seachrist DD Montañez-Wiscovich ME Danielpour D Keri RA. Gene expression profiling of cancer progression reveals intrinsic regulation of transforming growth factor-beta signaling in ErbB2/Neu-induced tumors from transgenic mice. Oncogene (2005) 24(33):5173–90. 10.1038/sj.onc.1208712 PMC1431507 15897883

[29] Sweet-Cordero A Mukherjee S Subramanian A You H Roix JJ Ladd-Acosta C. An oncogenic KRAS2 expression signature identified by cross-species gene-expression analysis. Nat Genet (2005) 37(1):48–55. 10.1038/ng1490 15608639

[30] Hummerich L Müller R Hess J Kokocinski F Hahn M Fürstenberger G. Identification of novel tumour-associated genes differentially expressed in the process of squamous cell cancer development. Oncogene (2006) 25(1):111–21. 10.1038/sj.onc.1209016 16247483

[31] Mikkelsen TS Hanna J Zhang X Ku M Wernig M Schorderet P. Dissecting direct reprogramming through integrative genomic analysis. Nature (2008) 454(7200):49–55. 10.1038/nature07056 18509334 PMC2754827

[32] Cheng F Guo D. MET in glioma: signaling pathways and targeted therapies. J Exp Clin Cancer Res (2019) 38(1):270. 10.1186/s13046-019-1269-x 31221203 PMC6585013

[33] Xiang C Chen J Fu P. HGF/Met Signaling in Cancer Invasion: The Impact on Cytoskeleton Remodeling. Cancers (Basel) (2017) 9(5):44. 10.3390/cancers9050044 PMC5447954 28475121

[34] Luo MS Huang GJ Liu BX. Immune infiltration in nasopharyngeal carcinoma based on gene expression. Medicine (Baltimore) (2019) 98(39):e17311. 10.1097/MD.0000000000017311 31574860 PMC6775332

[35] Meng J Liu Y Guan S Fan S Zhou J Zhang M. The establishment of immune infiltration based novel recurrence predicting nomogram in prostate cancer. Cancer Med (2019) 8(11):5202–13. 10.1002/cam4.2433 PMC6718526 31355524

[36] Ge P Wang W Li L Zhang G Gao Z Tang Z. Profiles of immune cell infiltration and immune-related genes in the tumor microenvironment of colorectal cancer. Biomed Pharmacother (2019) 118:109228. 10.1016/j.biopha.2019.109228 31351430

[37] Fristedt R Borg D Hedner C Berntsson J Nodin B Eberhard J. Prognostic impact of tumour-associated B cells and plasma cells in oesophageal and gastric adenocarcinoma. J Gastrointest Oncol (2016) 7(6):848–59. 10.21037/jgo.2016.11.07 PMC5177573 28078109

[38] Spear S Candido JB McDermott JR Ghirelli C Maniati E Beers SA. Discrepancies in the Tumor Microenvironment of Spontaneous and Orthotopic Murine Models of Pancreatic Cancer Uncover a New Immunostimulatory Phenotype for B Cells. Front Immunol (2019) 10:542. 10.3389/fimmu.2019.00542 30972056 PMC6445859

[39] Shi JY Gao Q Wang ZC Zhou J Wang XY Min ZH. Margin-infiltrating CD20(+) B cells display an atypical memory phenotype and correlate with favorable prognosis in hepatocellular carcinoma. Clin Cancer Res (2013) 19(21):5994–6005. 10.1158/1078-0432.CCR-12-3497 24056784

[40] Nielsen JS Sahota RA Milne K Kost SE Nesslinger NJ Watson PH. CD20+ tumor-infiltrating lymphocytes have an atypical CD27- memory phenotype and together with CD8+ T cells promote favorable prognosis in ovarian cancer. Clin Cancer Res (2012) 18(12):3281–92. 10.1158/1078-0432.CCR-12-0234 22553348

[41] Yamane H Paul WE. Cytokines of the γ(c) family control CD4+ T cell differentiation and function. Nat Immunol (2012) 13(11):1037–44. 10.1038/ni.2431 PMC4825860 23080204

[42] Fridman WH Pagès F Sautès-Fridman C Galon J. The immune contexture in human tumours: impact on clinical outcome. Nat Rev Cancer (2012) 12(4):298–306. 10.1038/nrc3245 22419253

[43] Péguillet I Milder M Louis D Vincent-Salomon A Dorval T Piperno-Neumann S. High numbers of differentiated effector CD4 T cells are found in patients with cancer and correlate with clinical response after neoadjuvant therapy of breast cancer. Cancer Res (2014) 74(8):2204–16. 10.1158/0008-5472.CAN-13-2269 24535711

[44] Zhang Q Jia Q Deng T Song B Li L. Heterogeneous expansion of CD4+ tumor-infiltrating T-lymphocytes in clear cell renal cell carcinomas. Biochem Biophys Res Commun (2015) 458(1):70–6. 10.1016/j.bbrc.2015.01.069 25637538

