# Supplementary figures and images for: FAT3 Mutation Is Associated With Tumor Mutation Burden and Poor Prognosis in Esophageal Cancer
Source: Front Oncol. 2021 Mar 19;11:603660. doi: 10.3389/fonc.2021.603660 (PMC8018597; doi:10.3389/fonc.2021.603660)

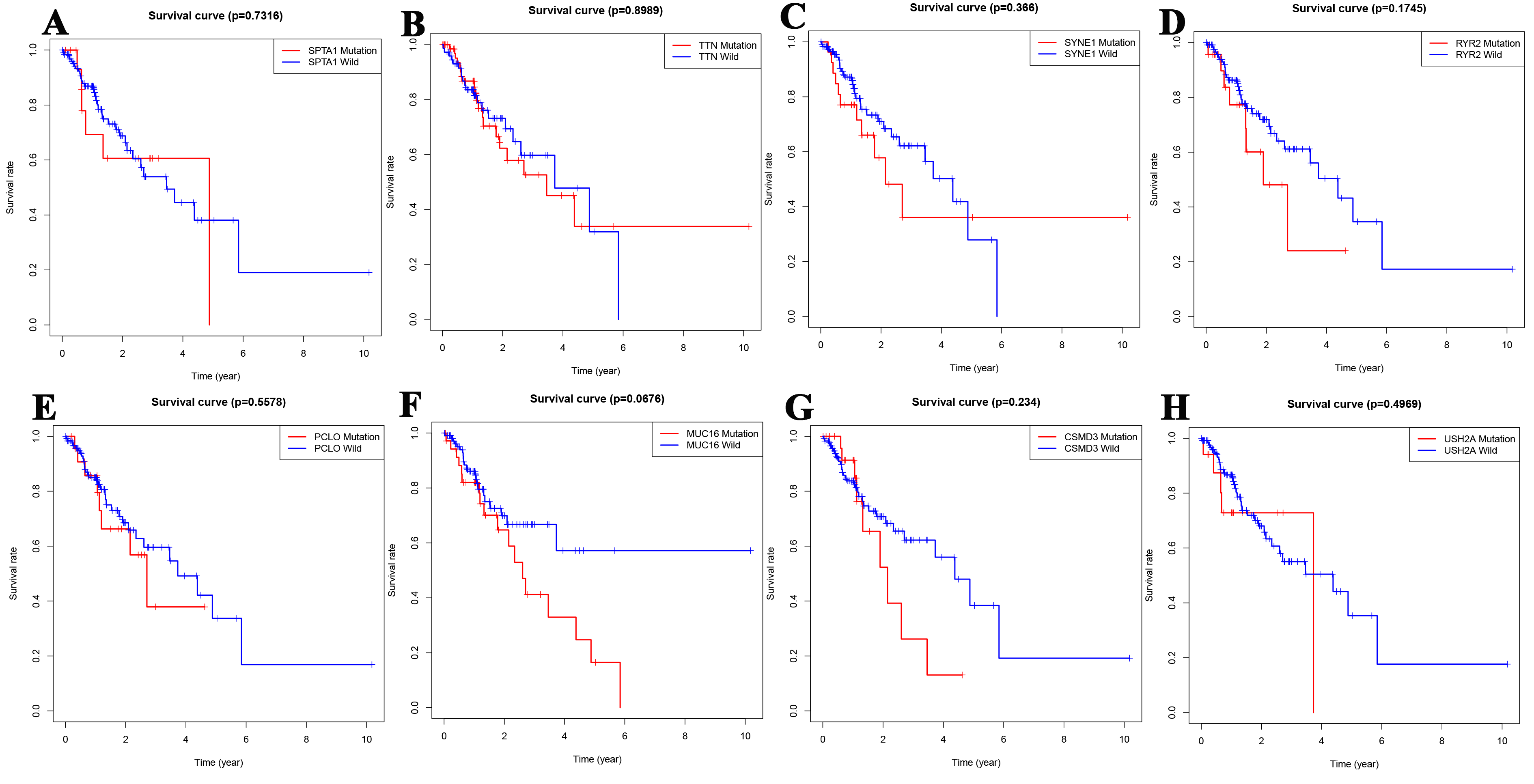

Supplement: Supplementary Figure 1 — Gene mutations aren’t associated with clinical prognosis. (A–H) The association of gene mutations with survival prognosis was analyzed by Kaplan-Meier method. A total of 144 samples containing complete clinical information were included. P-values<0.05 were considered significant. WT, wild type; MT, mutant type. [file Image_1.tif]
